# Scaling of the AIS and Somatodendritic Compartments in α S RGCs

**DOI:** 10.3389/fncel.2019.00436

**Published:** 2019-09-27

**Authors:** Vineeth Raghuram, Paul Werginz, Shelley I. Fried

**Affiliations:** ^1^Rehabilitation Research & Development Service, Boston VA Healthcare System, Boston, MA, United States; ^2^Department of Neurosurgery, Massachusetts General Hospital, Harvard Medical School, Boston, MA, United States; ^3^Department of Biomedical Engineering, Tufts University, Medford, MA, United States; ^4^Institute for Analysis and Scientific Computing, Vienna University of Technology, Vienna, Austria

**Keywords:** axon initial segment, retinal ganglion cells, morphological scaling, axonal geometry, action potential

## Abstract

The anatomical properties of the axon initial segment (AIS) are tailored in certain types of CNS neurons to help optimize different aspects of neuronal function. Here, we questioned whether the AISs of retinal ganglion cells (RGC) were similarly customized, and if so, whether they supported specific RGC functions. To explore this, we measured the AIS properties in alpha sustained RGCs (α S RGCs) of mouse; α S RGCs sizes vary systematically along the nasal temporal axis of the retina, making these cells an attractive population with which to study potential correlations between AIS properties and cell size. Measurements of AIS length as well as distance from the soma revealed that both were scaled to cell size, i.e., cells with large dendritic fields had long AISs that were relatively far from the soma. Within the AIS, the percentage of Na_*v*_1.6 voltage-gated sodium channels remained highly consistent, regardless of cell size or other AIS properties. Although ON RGCs were slightly larger than OFF cells at any given location of the retina, the level of scaling and relative distribution of voltage-gated sodium channels were highly similar. Computational modeling revealed that AIS scaling influenced spiking thresholds, spike rate as well as the kinetics of individual action potentials, Interestingly, the effect of individual features of the AIS varied for different neuronal functions, e.g., AIS length had a larger effect on the efficacy by which the AIS initiated spike triggered the somatic spike than it did on repetitive spiking. The polarity of the effect varied for different properties, i.e., increases to soma size increased spike threshold while increases to AIS length decreased threshold. Thus, variations in the relative level of scaling for individual components could fine tune threshold or other neuronal functions. Light responses were highly consistent across the full range of cell sizes suggesting that scaling may post-synaptically shape response stability, e.g., in addition to several well-known pre-synaptic contributors.

## Introduction

The axon initial segment (AIS) is a specialized portion of the proximal axon that mediates the initiation and propagation of action potentials in CNS neurons. Structurally, the AIS is comprised of densely packed voltage-gated sodium (Na_*v*_) and potassium channels (K_*v*_) that are held in place by a complex network of structural proteins and tethering molecules. Much recent evidence suggests that AISs are tailored to help optimize specific elements of neuronal function. For example, neurons of the chick nucleus laminaris that are sensitive to low auditory frequencies have AISs that are shorter and further from the soma than the AISs of high-frequency neurons; the differences in the length and location are thought to help maximize the sensitivity to the interaural time differences (ITD) associated with each frequency range (Kuba et al., 2006). In contrast, the length of the AIS does not vary systematically in thick-tufted pyramidal neurons of the somatosensory cortex but instead, the distance between the AIS and the soma is inversely correlated to the size of the apical dendrite; this arrangement helps maintain consistency in the amplitude of the back-propagated action potential across a wide range of cellular morphologies (Hamada et al., 2016).

Within the AIS, two spatially and functionally distinct sub-types of voltage-gated sodium channels are thought to play complementary roles in the generation and back-propagation of spiking (Boiko et al., 2003; Van Wart et al., 2007; Hu et al., 2009). Na_*v*_1.6 channels are confined to the distal portion of the AIS and activate at lower levels of membrane depolarization; consistent with their higher sensitivity and greater separation from the soma (i.e., more decoupled), spikes are initiated in this portion of the AIS. In contrast, Na_*v*_1.2 channels are confined to the proximal portion of the AIS and have higher activation thresholds; these channels remain largely unactivated by the initial depolarization of the soma and thus are well-suited to effectively facilitate penetration of the AIS-initiated spike into the soma. In a previous study in rat retinal ganglion cells (RGCs) (Van Wart et al., 2007) the Na_*v*_1.6 portion was reported to comprise ∼2/3 of the total AIS length although details were not provided as to whether the ratio was for a single cell type or an average across the population of all RGCs tested. Given the specificity of the AIS in different cell types, it seems likely that the composition of the AIS, e.g., the distribution of Na_*v*_ channels may also be tailored for individual cells or cell types.

Somewhat surprisingly, neither the structure nor the function of the AIS has been well studied in retinal neurons. Most mammalian retinas contain over 30 different types of ganglion cells (retinal output neurons); each type extracts different features of the visual world and uses distinct patterns of spiking to convey information to higher visual centers (Devries and Baylor, 1997; Roska and Werblin, 2001; Baden et al., 2016). Given the functional specificity of the AIS in other types of CNS neurons, it seems reasonable to question whether the AISs of individual RGC types are also customized to meet specific functional requirements. For example, the AISs of cell types that generate long-duration, high-frequency bursts of spikes may be different from the AISs of types that generate short-duration, low-frequency spike trains. Potential support for AIS specificity in RGCs comes from an earlier study that found longer AISs in brisk-transient (BT) vs. directionally selective (DS) RGCs of the rabbit retina (Fried et al., 2009). Surprisingly however, there was a great deal of overlap between the two populations with the result that the length of the AIS in many BT cells was actually shorter than that of some DS cells. This variability is somewhat curious and raises the possibility that the absolute length of the AIS may be less critical than its length relative to other cellular features.

The alpha Sustained (α S) RGCs of the mouse retina is a particularly attractive population with which to study AIS structure and function because the size of these cells varies systematically across the retina (Bleckert et al., 2014), i.e., the relationship between AIS properties and cell size can be directly evaluated. Further, because RGCs remain intact in the flat-mount preparation, AIS properties can be directly correlated to a wide range of other cellular features. The fact that α S RGCs consist of both ON and OFF sub-types (Pang et al., 2003) is also attractive because AIS properties can be compared across two, functionally similar populations. Finally, the light responses of α S RGCs provide a measure of neuronal function, allowing correlations between AIS structure and cellular function to be explored. Much previous work with the α S population has led to well characterized neuronal features that facilitate the rapid and unequivocal identification of targeted cells.

Here, we measured the anatomical properties of the AIS and of other cellular features in a large number of ON- and OFF-α S RGCs that spanned the full extent of the mouse retina. We found that many cellular features, including AIS length and location, varied systematically across the retina and were generally scaled to one another. The observed scaling was similar in both ON- and OFF-α S RGCs although ON cells were generally larger at any given retinal location. Unlike other cellular properties, the composition of the AIS was highly consistent across all cells but differed from previously reported values. The functional role(s) of the anatomical variations were explored using a series of morphologically- and biophysically realistic computational models; scaling was shown to influence neuronal sensitivity, spike rate and the effectiveness with which AIS initiated spikes trigger somatic spikes. Scaling may also help to stabilize responses across the population.

## Materials and Methods

### Immunohistochemistry

GFP line M (Thy-1 promoter) mice were chosen for anatomical experiments due to the sparse fluorescent labeling of individual RGC cell bodies as well as their complete dendritic trees (Feng et al., 2000), which enabled the ability to perform detailed morphological analyses (see below). Retinal whole-mounts were surgically isolated from Thy-1 GFP line M mice (Jackson Laboratory), fixed in 4% paraformaldehyde (w/v) solution for 30 min, washed in PBS, pH 7.4, for 1 h, and subsequently placed into 12-well clear multiwell plates (Corning Falcon) for processing through free-floating immunochemistry. Retinas were then blocked with 5% normal donkey serum (NDS) in 0.3% PBTX (10x PBS + Triton X) solution for 2 h, and then incubated with chicken anti-GFP (1/200; Abcam), mouse anti-AnkyrinG (1/200; NeuroMab clone N 106-36), rabbit anti-Na_v_1.6 (1/200; Millipore Sigma), and goat anti-ChAT (1/200; Millipore Sigma) in 1% NDS with 0.3% PBTX solution for 5 overnights. Following incubation in primaries, samples were washed in PBS for 2 h (4 × 30 min), and placed in Alexa Fluor 488-conjugated donkey anti-chicken (1:200; Jackson Immuno), Cy5-conjugated donkey anti-mouse (1:200; Jackson ImmunoResearch), Alexa Fluor 405-conjugated donkey anti-rabbit (1:200; Abcam), and Alexa Fluor 594-conjugated donkey anti-goat (1:200; Invitrogen) secondary antibodies in 0.3% PBTX solution for 1 overnight. Finally, samples were washed in PBS for 1 h (2 × 30 min), mounted with Prolong gold anti-fade mounting medium (Invitrogen), cover-slipped, and placed in 4°C refrigeration until further confocal imaging.

### Image Acquisition and Analysis

**FIGURE 1:**
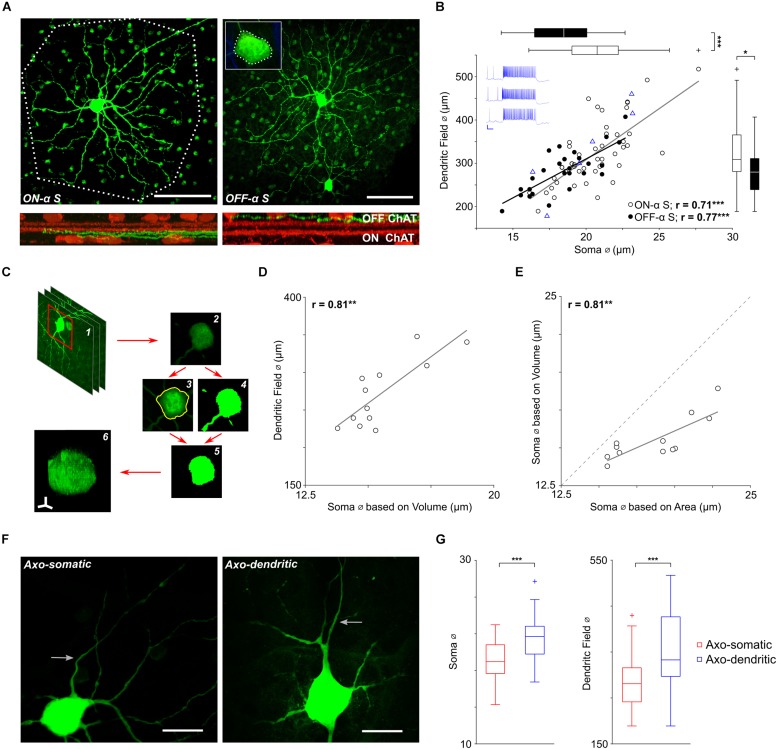
Soma size scales with dendritic field size in α S RGCs. (**A**, top) The soma and dendritic field of a typical ON-α S (left) and a typical OFF-α S (right) RGC from flat mount preparations of different Thy-1 GFP-M mice. Polygons drawn around the periphery of the ON cell’s terminal dendrites (dotted line) and the soma of the OFF cell (inset) were used to estimate the size of each (see text). Scale bars: 100 μm. (Bottom) Cross-sectional views of the confocal image stack reveal the stratification level of the terminal dendrites (green) relative to that of the ON and OFF ChAT bands (red). **(B)** Each point is a plot of soma diameter vs. dendritic field diameter for an individual ON- (unfilled) or OFF-α S RGC (filled). Blue triangles (*n* = 7) are from ON-α S cells for which light responses were also obtained; blue traces on the top left are representative light responses from 3 of the cells (Scale bar: 200 ms/20 mV). Gray and black lines are the best-fit linear regressions for ON- (*p* = 2 × 10-8) and OFF- (*p* = 5 × 10-6) α S cell populations, respectively. Box plots (right and top) reveal statistically significant differences in soma diameter (*p* = 2 × 10-4), and dendritic field diameter (*p* = 0. 0164) between ON- vs. OFF-α S RGCs. **(C)** The volume of the soma was calculated by (1,2) obtaining the raw image stack, (3) converting it into a binary image file, (4) using the maximum intensity projection to create a region of interest (ROI) around the cell body, (5) clearing the area outside of the ROI, (6) displaying the result in the NIH Image J/FIJI 3D viewer and using the voxel counter plugin. **(D)** Each point (*n* = 12) is a plot of the volume-based soma diameters were plotted vs. dendritic field diameter. The solid gray line is the best-fit linear regression (*p* = 0.0014). **(E)** Similar to D, each point is a plot of soma volume calculated from 2-d measurements vs. soma volume measured from 3-d reconstructions (see text). The solid grey line is the best-fit linear regression (*p* = 0.0016). **(F)** Expanded view of the soma and proximal axon region from two ON-α S RGCs reveals that axons (arrows) can emerge from the soma (left) or from a primary dendrite (right). Scale bars: 20 μm. **(G)** Box plots reveal statistically significant differences in soma diameter (*p* = 3 × 10-5), and dendritic field diameter (*p* = 8 × 10-5) for axo-somatic vs. axo-dendritic cells.

Fluorescence imaging was performed with a laser scanning confocal microscope (Zeiss LSM 880 with Airyscan) using Zeiss Efficient Navigation (ZEN Pro) software to acquire and export images. Images of the full RGC dendritic field morphology were acquired with 20x magnification at a x/y/z resolution of 0.15 × 0.15 × 0.40 μm, while images of the soma, as well as proximal axon with AIS labeling were acquired with a 63x oil immersion objective (NA = 1.4) at a x/y/z resolution of 0.09 × 0.09 × 0.40 μm. The use of a higher magnification (63x) as well as the Thy-1 line M GFP mouse (Feng et al., 2000), which provides complete labeling of RGC morphologies, allowed for the ability to perform precise tracing around the cell body and dendritic tree. Anatomical measurements and image post-processing of confocal scans were performed in NIH Image J/FIJI package (Schindelin et al., 2012). Calculation of cell volume was performed in NIH Image J/FIJI by first converting the raw image stack into a binary image (63x magnification) using median filters and local auto thresholding via the Bernsen method (Figure 1C) (Bernsen, 1986); additional image processing was performed using the NIH Image J/FIJI binary processing toolbox. Separately, a region of interest (ROI) is determined by creating a maximum intensity projection of the cell in the z-dimension and then manually tracing a contour around the cell body. The ROI is then applied to the processed binary image file, and the area outside the cell body is cleared to isolate the soma. This cropped binary image of the soma is then displayed in the 3D viewer (Schmid et al., 2010) where the cell volume is calculated using the voxel counter plugin.

Confirmation of cell type was obtained using choline acetyl-transferase (ChAT), an immunochemical marker which labels the processes of ON and OFF starburst amacrine cells (Baughman and Bader, 1977). These are otherwise referred to as (ON- and OFF-) ChAT bands and can be used to delineate the specific sublamina in which the terminal dendrites of tentatively identified RGCs stratified (Jeon et al., 1998). Consistent with previous reports, the terminal dendrites of putative ON and OFF-α S RGCs stratified above and below the ON- and OFF-ChAT bands (van Wyk et al., 2009).

The anatomical properties (e.g., start and end position, distance from the soma, length) of the AIS in confocal image stacks (63x magnification) were measured by manually identifying and precisely tracing along the region of colocalization between Ankyrin_G_/Na_v_1.6 and the green fluorescent signal of the native GFP expressed within the axons of labeled RGCs using NIH Image J/FIJI package. Measurements of AIS length and distance were performed independently from calculations of cell size (dendritic field diameter, soma diameter, soma volume). Measurements were reliable between 3 independent experimenters. In a few cases (<10), AISs were obscured, either because they overlapped with other AISs or because of interfering axon bundles, making it difficult to obtain an accurate measurement and to get agreement between examiners. In these cases, the AIS measurements were excluded from further analysis.

Simple 3-d tracing of dendritic architecture and Sholl analysis was performed in NIH ImageJ/FIJI toolbox Simple Neurite Tracer (Longair et al., 2011). Full tracing, including compartment diameters of dendritic arbors, was performed in NeuronStudio (20x magnification) (Wearne et al., 2005). Tracing of diameters of the proximal axon was performed at higher magnification (63x).

### Electrophysiology

The care and use of animals followed all federal and institutional guidelines and all protocols were approved by the Institutional Animal Care and Use Committee (IACUC) of the Massachusetts General Hospital. Wild type (C57BL/6J) mice (Charles River Laboratories) were anesthetized with isofluorane (Henry Schein) and subsequently euthanized by cervical dislocation. Eyeballs were harvested, retinas were dissected from the eyecup and mounted, photoreceptor side down, onto a recording chamber. The retina was subsequently perfused with oxygenated Ames medium (Sigma-Aldrich) buffered to pH 7.4 at a flow rate of 2–3 ml/min for the duration of the experiment. Temperature was maintained at ∼34°C. Small holes were made in the inner limiting membrane in order to obtain access to RGC somata. Spiking responses were obtained using loose or whole cell patch recordings. Intracellular solution consisted of (in mM, all Sigma-Aldrich): 125 K-gluconate, 10 KCl, 10 Hepes, 10 EGTA, 4 Mg-ATP, 1 Na-GTP. Morphological analysis was allowed by adding Neurobiotin (0.25 mM, Vector Laboratories) and Alexa 488 (0.5%, Invitrogen) to the intracellular solution. Further processing of filled RGCs was performed as described above (Immunohistochemistry), but primary/secondary incubation to recover cell morphology was done with Alexa Fluor 488-conjugated streptavidin (1:200; Invitrogen).

Visual stimulation consisted of bright spots on neutral (gray) background with diameters ranging from 100–1500 μm and presented for 1 s. ON-α S cells were targeted by their large somata (diameter > 15 μm) and identified by their strong sustained light responses (Pang et al., 2003; van Wyk et al., 2009; Krieger et al., 2017).

Stimulus control and data acquisition were performed with custom software written in LabView (National Instruments) and Matlab (Mathworks). Data were recorded using an Axopatch 700B amplifier (Molecular Devices) and digitized by a data acquisition card (PCI-MIO-16E-4, National Instruments). The timing of individual spikes was detected as the depolarization (loose patch = negative; whole cell patch = positive) peak of each spike in the raw trace. Firing rate was computed by pooling responses from multiple trials (≥3) and subsequent convolution with a 50 ms sliding window.

### Computational Modeling

**FIGURE 2:**
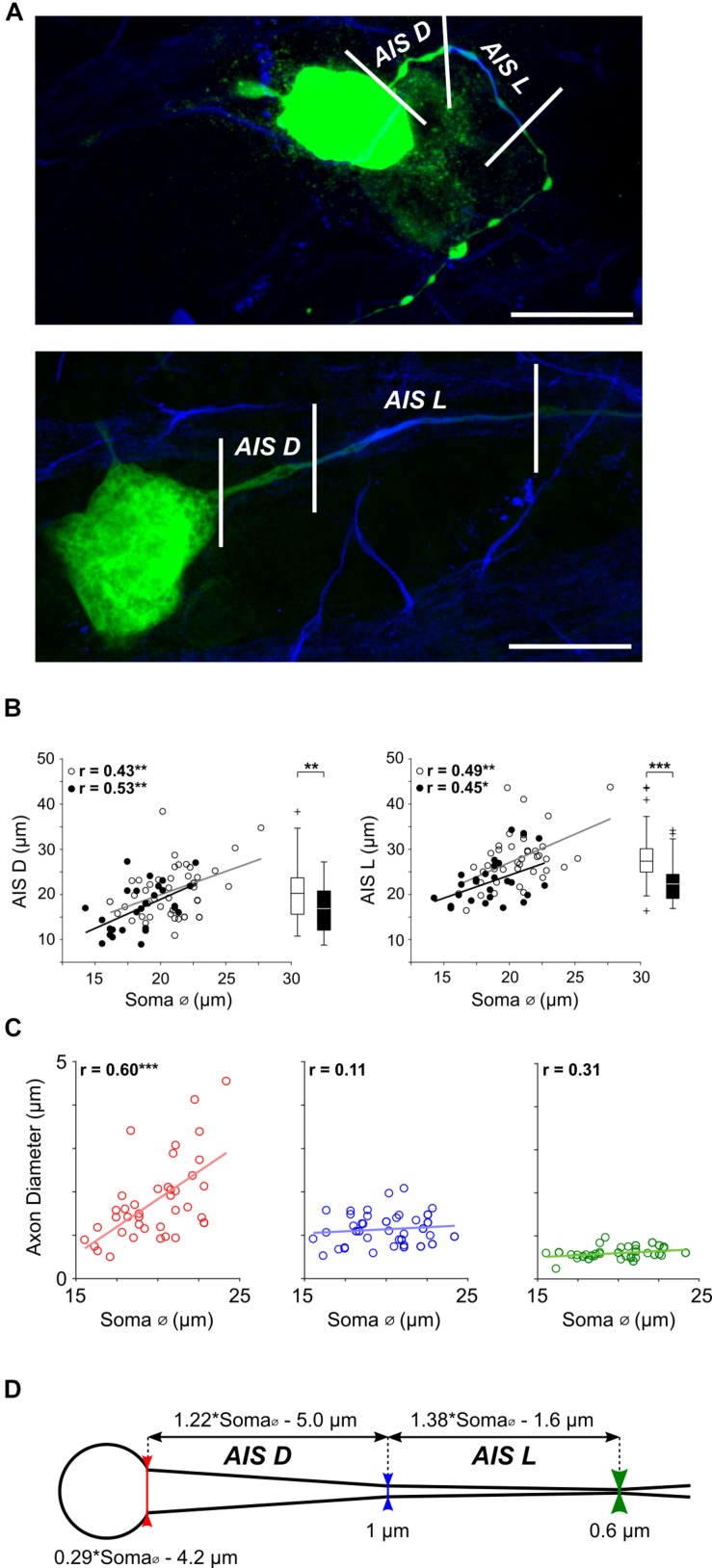
AIS features scale with cell size in α S RGCs. **(A)** Expanded view of the soma and proximal axon region from two typical ON-α S RGC; a small RGC with a soma diameter of ∼17 μm (top) and a large RGC with a soma diameter of ∼24 μm (bottom). Staining for Ankyrin_G_ (blue) was used as a marker for the AIS and allowed both length (AIS L, distance between the middle and right vertical lines), as well as distance from the soma (AIS D, distance between the left and middle lines) to be determined. Scale bars: 20 μm. **(B)** Each point is a plot of soma diameter vs. AIS distance (left) or soma diameter vs. AIS length (right), for individual ON- (unfilled) or OFF-α S RGCs (filled). Gray and black lines are the best-fit linear regressions for ON (AIS D: *p* = 0.0045, AIS L: *p* = 0.0015) and OFF (AIS D: *p* = 0.0043, (AIS L: *p* = 0.0220) α S cell populations, respectively. Box plots (right) reveal statistically significant differences in AIS D (left, *p* = 0.0078), and AIS L (right, *p* = 7 × 10-4) for ON vs. OFF cells. **(C)** The diameter of the proximal axon was measured at the soma-axon border [red vertical line in **(D)**], the proximal edge of the AIS (blue vertical line) and the distal edge of the AIS (green vertical line) in 39 α S RGCs. Dashed lines indicate best fit linear regressions. The diameter of the axon varied with cell size at the soma-axon border (red, *p* = 0.0001) but not at the other two locations (*p* = 0.5012 and *p* = 0.0541, respectively). **(D)** Schematic representation of the soma and proximal axon of an α S RGC depicting the relationship between AIS properties and soma diameter; equations at top were derived from the best-fit plots in **(B)**.)

To simulate the response of model RGCs to somatic current injection we used the approach of compartment modeling, similar to much previous work from our group (Jeng et al., 2011; Werginz et al., 2014). Thereby, current flow along the stimulated fiber as well as current flow across the neuronal membrane is simulated by a network of compartments with given electrical (membrane) properties. We used membrane dynamics from Fohlmeister (Fohlmeister et al., 2010) and ion channel densities for the five different sections along the neuron [dendrites, soma, axon hillock (soma to AIS), AIS, axon] can be found in Supplementary Table S1. Consistent with previous work in the retina (Jeng et al., 2011; Guo et al., 2013), the levels of voltage-gated sodium and potassium ion channels in the proximal axon were approximately 7x greater than those in the soma. Intracellular (axial) resistivity was set to 143.2 Ω⋅cm (Fohlmeister et al., 2010), specific membrane capacitance 1 μF/cm^2^ and model temperature was set to 35°C similar to experimental conditions. The model was solved in Matlab (Mathworks) using a custom written implicit (backward) Euler solver. Time step was set to 0.005 ms. Cell morphologies of traced cells consisted of 1388–2455 compartments depending on cell size; compartment length ranged from 3 to 5 μm. Axonal geometries were reconstructed based on trendlines derived from anatomical measurements (Figure 2D).

Single-spike thresholds were determined as the current amplitude necessary to elicit an action potential by a 1 ms rectangular current injection. Action potentials were detected within 3 ms after pulse offset. Spike trains were generated by constant current injections (100 pA) 400 ms in length.

### Statistical Analysis

We measured linear correlation between two variables using Pearson’s correlation coefficient. For comparison of means, we used a two-sample *t*-test. Significance levels were set as follows: n.s. *p* ≥ 0.05, ^∗^*p* < 0.05, ^∗∗^*p* < 0.01, ^∗∗∗^*p* < 0.001. Numerical values are presented as mean ± standard deviation of mean (SD). Boxplots use standard notation (1st Quartile, Median, 3rd Quartile); outliers are indicated outside 1.5x the inter-quartile range (3rd Quartile - 1st Quartile). All statistical analysis was performed in Matlab (Mathworks).

## Results

### α S RGCs Have Readily Identifiable Anatomical Properties

The sparse labeling of the GFP M mouse model (Feng et al., 2000) allowed us to tentatively identify α S RGCs based on previously described morphological features (Völgyi et al., 2009; Baden et al., 2016; Bae et al., 2018) such as soma size >15 μm and dendritic field diameter >200 μm (Figure 1A, top panels). Total dendritic length, computation of mean segment length and Sholl analysis (Sholl, 1953) in presumed α S RGCs were also highly similar to previous reports (Bleckert et al., 2014) (Supplementary Figure S1), providing additional confirmation of cell type. Final verification was obtained from the stratification level of terminal dendrites (van Wyk et al., 2009): ON-α S RGCs stratified just below the ON-ChAT band (Figure 1A bottom, left) while OFF cells stratified just above the OFF-ChAT band (Figure 1A bottom, right). Using this approach, we identified a total of 47 ON-α S and 27 OFF-α S RGCs obtained from 11 different mice. Light responses were measured in seven additional putative ON-α S RGCs (Figure 1B, top left, blue traces) and found to be highly similar to those described previously (Pang et al., 2003; van Wyk et al., 2009; Krieger et al., 2017). Subsequent morphological analysis of these seven additional cells, using the approach described above, were consistent with the findings from the original 47 ON cells, adding additional support to the notion that the cells studied here were indeed the same population of ON-α S RGCs described in previous anatomical and physiological studies (Pang et al., 2003; van Wyk et al., 2009; Krieger et al., 2017).

Consistent with earlier studies (Bleckert et al., 2014), the spatial extent of the dendritic field was quantified by drawing a polygon around the edges of the cell’s terminal dendrites (Figure 1A, left) and then calculating the diameter of the circle whose area matched that of the drawn polygon. A similar approach was also used to calculate the diameter of the soma (Figure 1A, inset), but unlike prior studies (Bleckert et al., 2014) which used maximum intensity projections at low magnification (20x) to estimate the perimeter of the soma, we were able to more clearly delineate the boundaries by manually tracing the cell body along the entire z-dimension using high (63x) magnification (see section Materials and Methods). We plotted soma size vs. dendritic field diameter for all ON- and OFF-α S cells (Figure 1B), unfilled (*n* = 47) and filled (*n* = 27), respectively; blue triangles represent the 7 ON cells for which light responses were also obtained). Calculating the best-fit linear regressions for the ON and OFF populations (gray and black lines, respectively) revealed a highly significant positive correlation in both populations (ON: *p* = 2 × 10-8; OFF: *p* = 5 × 10-6), suggesting that the size of the dendritic field scales with the size of the soma. This correlation differs from the findings of an earlier study with this same RGC population in which the size of the soma did not vary systematically with the size of the dendritic field (Bleckert et al., 2014). While the difference in methodology is likely to contribute to the discrepancy, we nevertheless sought to more precisely estimate the soma size of each cell using numerical integration. Briefly, we determined the somatic voxel contribution from each individual confocal slice and then summed the contribution from all slices to estimate the total somatic ‘3-d’ volume (Figure 1C, see section Materials and Methods). The calculated diameter of the soma from the 3-d cell volume (*n* = 12) was highly correlated to its dendritic field size (Figure 1D), providing additional support that soma size does indeed scale with cell size. Comparison of the soma diameter calculated using the 3-d volume to the soma diameter estimated from the 2-d projection across the z-stack (Figure 1E) suggested the 2-d approach overestimates the somatic volume and is consistent with our observation that most α S somas appeared ‘flattened,’ i.e., not perfectly spherical (Bae et al., 2018). The linear correlation between the two estimates (Figure 1E) nevertheless suggests that soma size does increase with dendritic field size across the entire population of α S RGCs, i.e., not just the 12 cells for which were able to perform the full 3-d estimates.

The finding that soma size is correlated to dendritic field size in α S RGCs of mouse is consistent with previous reports in other types of RGCs, including α RGCs of rabbit retina (Peichl et al., 1987) as well as midget and parasol RGCs of the primate retina (Watanabe and Rodieck, 1989). This type of scaling may be cell type specific as there were significant variations in dendritic field diameter but not soma diameter in dorsal vs. ventral OFF-alpha transient (α T) RGCs of the mouse (Warwick et al., 2018). Both the size of the soma as well as the size of the dendritic field were smaller in OFF-α S RGCs than in neighboring ON cells (Supplementary Figure S2 and Figure 1B, box plots at right and top, respectively), consistent with previous reports in both human and primate RGCs (Dacey and Petersen, 1992; Chichilnisky and Kalmar, 2002).

We also measured the diameters of the dendritic sections from the 7 filled ON-α S RGCs to see if they too were correlated to cell size (see section Materials and Methods). A histogram of the dendritic diameters (Supplementary Figure S3A) revealed that 95% were between 0.36 μm and 1.48 μm (mean of 0.92 μm). A probability distribution function (PDF) was fit to each histogram (red trace) and then PDFs from all cells were overlaid (Supplementary Figure S3B, red lines); the distribution of dendritic diameters was similar for all cells, i.e., it was not dependent on cell size. Consequently, total dendritic surface area was found to scale linearly with dendritic field diameter (Supplementary Figure S3C, *p* = 0.0028).

In most α S RGCs (*n* = 50/74) the axon emerged directly from the soma, but in other cells, it emerged instead from a primary dendrite (Figure 1F, compare left and right panels). This arrangement is similar to that reported in other types of CNS neurons (Hamada et al., 2016; Höfflin et al., 2017) although in some of these other types, axons can emerge from secondary or even tertiary dendritic processes while here, axo-dendritic axons emerged only from primary dendrites. α S RGCs with axo-somatic axons tended to be smaller than RGCs with axo-dendritic axons (mean soma diameters of 19.1 vs. 21.6 μm, *p* = 3 × 10-5, and mean dendritic field diameter of 287 vs. 354 μm, *p* = 8 × 10-5) (Figure 1G). Most axo-dendritic α S RGCs were ON cells (*n* = 21/24).

### AIS Properties Also Scale With Cell Size

The AIS is typically the site of spike initiation in CNS neurons (Stuart et al., 1997). The high density of voltage-gated sodium channels in this region (Kole et al., 2008) combined with its much smaller surface area (vs. that of the soma) leads to more rapid depolarization of membrane voltage with a corresponding earlier onset of spiking, even though excitatory synaptic inputs typically result in a slightly stronger initial depolarization at the soma. Both the size as well as the location of the AIS can influence neuronal function (Kuba et al., 2006; Evans et al., 2015; Hamada et al., 2016) although interestingly, AIS properties mediate distinct functions in different types of neurons. For example, the length and location of the AIS is customized in individual neurons of the nucleus laminaris to help optimize sensitivity to specific auditory frequencies (Kuba et al., 2006) while the location of the AIS is tailored in thick-tufted L5 pyramidal neurons to help normalize spike amplitude across a wide range of cell sizes (Hamada et al., 2016).

Given the importance of AIS properties in shaping neuronal output in other types of CNS neurons, we questioned whether AIS properties might similarly influence neuronal function in RGCs. If so, we hypothesized that similar to other CNS neurons, one or more features of the AIS would be correlated to other morphological features of the cell; the systematic variations in cell size across the α S population (Figure 1 and Supplementary Figure S2) provide an ideal substrate from which to explore this hypothesis. Variability in both the length and the location of the AIS has been reported previously in two types of rabbit RGCs (Fried et al., 2009) although any correlation between AIS properties and other RGC features was not reported in this earlier work.

Similar to previous approaches (Boiko et al., 2003; Van Wart et al., 2007; Fried et al., 2009) we used immunochemical labeling of Ankyrin_G_, a cytoskeletal anchoring protein (Kordeli et al., 1995) that enables dense packing of voltage-gated sodium channels in the AIS (Zhou et al., 1998), as a marker for the size and location of the AIS. The length of Ankyrin_G_ staining (Figure 2A, distance between middle and right vertical lines), hereafter referred to as AIS length or AIS L, did indeed vary considerably across the population of α S RGCs (Figure 2B, right). Further, the length of the AIS displayed a highly significant positive correlation to soma diameter (Figure 2B, right, unfilled and filled circles for ON and OFF RGCs, respectively; *p* = 1 × 10-6). The distance from the soma to the AIS, hereafter referred to as AIS distance or AIS D (Figure 2A, distance between left and middle lines), also varied across the population of α S RGCs with a similarly strong correlation to soma size (Figure 2B, left, *p* = 2 × 10-6). A comparison between the slopes of the best fit lines suggested that AIS length increases with soma size at a slightly faster rate (1.38 μm for every 1 μm increase in soma diameter) than does AIS distance (1.22 μm for every 1 μm increase in soma diameter); the equations describing the rates of increase are indicated above the schematic in Figure 2D.

In addition to the systematic scaling of AIS size and location, careful observation revealed variability in the diameter of the proximal edge of the axon. To quantify this, we measured the diameter of the proximal axon at three locations (Figures 2C,D, see section Materials and Methods): (1) the border between the soma and axon (Figure 2D, bottom, red vertical line), (2) the proximal edge of the AIS (blue line) and (3) the distal edge of the AIS (green line). Linear regressions of the measured points (*n* = 39) revealed that the diameter at the start of the axon was indeed correlated to soma size (Figure 2C, left, red, *p* = 0.0001) while the diameter at the proximal and distal ends of the AIS were not (Figure 2C, middle, blue, *p* = 0.5012; Figure 2C, right, green, *p* = 0.0541); diameters at these two locations were ∼0.6 and 1.0 μm, respectively. At locations >100 μm from the soma, the diameter of the distal axon remained largely constant at ∼1 μm, consistent with previous results in amphibian retina (Carras et al., 1992). Thus, consistent with the scaling of other neuronal features found earlier, the diameter of the proximal-most portion of the axon is also wider in larger AIS α S RGCs. Further, because the diameter of more distal portions of the axon does not scale with cell size, the angular taper between the AIS and the soma is also wider in large cells. Previous modeling work in neocortical pyramidal neurons found that the degree of angular taper in this region can influence the efficacy with which the AIS-initiated spike invades the soma (Mainen et al., 1995), suggesting a functional role for this variability (see below).

### The Ratio of Na_v_ Channel Sub-Components Remains Constant in α S RGCs

**FIGURE 3:**
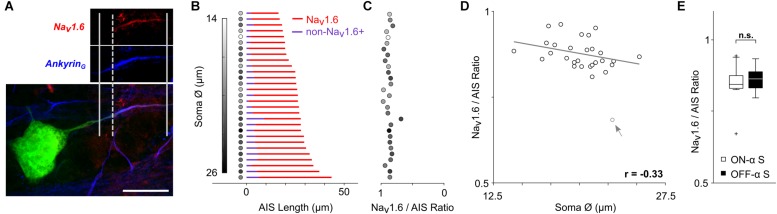
The relative composition of the AIS remains constant. **(A)** Staining for Ankyrin_G_ (blue) was used to capture the length of the AIS (distance between solid vertical lines); staining for Na_v_1.6 (red) was used to determine the length of the Na_v_1.6 component (distance between the rightmost solid and dashed vertical lines). Scale bar: 20 μm. **(B)** Each row corresponds to a single ON- or OFF-α S cell and depicts the composition of the AIS. The length of the red portion of the line corresponds to the length of the Na_v_1.6 component. The length of the blue portion (non-Na_v_1.6+) corresponds to the portion stained by Ankyrin_G_ but not by Na_v_1.6 and therefore likely to be the Na_v_1.1 component (see text). The shading of the circle to the left of each line corresponds to the size of the soma diameter for that cell (scale at left). **(C)** The calculated ratio of Na_v_1.6 length to total AIS (Ankyrin_G_) length for each cell (scale at bottom); the shading of filled circles is the same as in **(B)**. **(D)** Scatterplot of soma size vs. Na_v_1.6/AIS ratio (*p* = 0.0817). The gray arrow/data point indicates an outlier which was not used in correlation analysis. **(E)** Box plot reveals no statistically significant differences in soma diameter (*p* = 0.9366).

The AIS is comprised of two distinct sodium channel sub-types (Boiko et al., 2003; Van Wart et al., 2007; Hu et al., 2009): Na_v_1.6 channels cluster at the distal end of the AIS and have a relatively low activation threshold while Na_v_1.2 channels cluster at the proximal end and have a higher activation threshold (Hu et al., 2009). Note that in the retina, Na_v_1.1 channels are found in the proximal axon (Van Wart et al., 2007). Action potential initiation occurs in the Na_v_1.6 (low threshold) portion of the AIS (Hu et al., 2009; Kole and Stuart, 2012) while the lower sensitivity of the Na_v_1.2 portion is thought to facilitate the effectiveness with which the AIS-initiated spike triggers a somatic spike and may also regulate the amplitude of the back-propagated (somatic) spike (Hu et al., 2009). Given the distinct functional role of each sodium channel sub-type, we questioned whether their relative distribution within the AIS might also vary across the population of α S RGCs. Antibodies specific to Na_v_1.6 channels have been characterized previously in unclassified rodent RGCs (Van Wart et al., 2007; Damiani et al., 2012) as well as in other types of CNS neurons (Boiko et al., 2003; Hu et al., 2009), and were used here to selectively stain this portion of the AIS in α S RGCs (Figure 3A, see section Materials and Methods). Although we could not selectively stain the Na_v_1.1 component directly (but see Van Wart et al., 2007), the Na_v_1.1 and Na_v_1.6 components do not overlap (Boiko et al., 2003; Van Wart et al., 2007) and thus the length of the non-Na_v_1.6 + (presumed to be Na_v_1.1) component could be estimated by subtracting the Na_v_1.6 component from the length of the entire AIS. Figure 3B shows the distribution of each of the two sub-types of sodium channels across the population of all α S RGCs tested (*n* = 29). The red portion of each horizontal line corresponds to the measured length of the Na_v_1.6 component while the blue portion corresponds to the calculated length of the non-Na_v_1.6 + region component (the length of the Na_v_1.6 component subtracted from the length of the Ankyrin_G_ staining). The ratio of Na_v_1.6 length to total AIS length was calculated for each cell and ranged narrowly around a mean of 0.88 ± 0.04 (Figure 3C, range 0.81–0.96 with one outlier at 0.68). Plotting soma size vs. the Na_v_1.6/AIS ratio revealed a weak and slightly negative correlation that was not significant (Figure 3D, *p* = 0.0817). The ratio did not co-vary with any other morphological property of the cell (not shown).

The mean ratio found here (0.88) is higher than the two-thirds value described previously for unclassified types of RGCs in the rat retina (Van Wart et al., 2007). Few quantification details are provided in the earlier study and thus it is difficult to reach any definitive conclusions regarding the discrepancy in AIS composition between the two studies. It is possible of course that differences in species and/or cell-type contribute to the variability, but further work will be required to elucidate the actual reason(s). Nevertheless, the high level of consistency in AIS composition across the large number of α S RGCs studied here suggests that the relative proportion of channel sub-types may be essential for optimizing one or more functions of the AIS. There was no significant difference between the ratios for ON- vs. OFF-α S RGCs (Figure 3E, ON: 0.88 ± 0.04, range 0.81–0.96; OFF: 0.88 ± 0.04, range 0.81–0.95; *p* = 0.9366).

### Light Responses Are Independent of Cell Size

**FIGURE 4:**
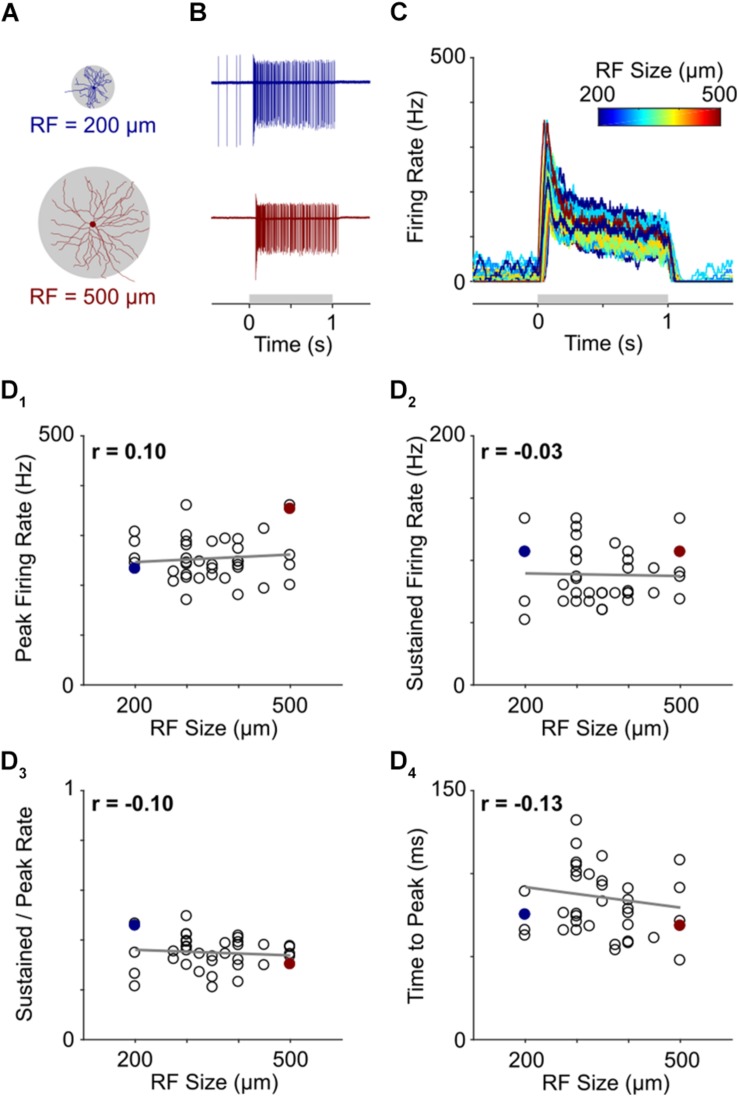
Light responses are independent of RGC size. **(A)** Representative morphological traces of two ON-α S RGCs from temporal (top) and nasal (bottom) retina. **(B)** Spiking responses from a small cell (top) and large cell (bottom) to a 1 s light flash. The spot sizes were 200 and 500 μm for the top and bottom cells, respectively, and corresponded to the stimulus size that produced the highest peak firing rate in each cell. The gray horizontal bar (bottom) represents the duration of the stimulus. **(C)** Overlay of firing rate vs. time for 39 ON-α S RGCs. For each cell, the response presented is to the spot size that generated the strongest spiking responses (range: 200–500 μm); line color indicates the size of the flashed spot that produced the peak response (Scale at top). **(D)** Peak firing rate **(D_1_)**, sustained firing rate **(D_2_)**, sustained firing rate divided by peak firing rate **(D_3_)** and time to peak **(D_4_)** are all plotted as a function of receptive field size for all cells tested. None of the correlations were found to be significant (*p* = 0.5557, *p* = 0.8660, *p* = 0.5438, and *p* = 0.4263, respectively). The two cells from **(B)** are highlighted by filled markers.

The wide range of morphological variability across α S RGCs raises the question as to whether light responses would remain consistent across such a wide variation. Previous studies have shown that light responses from nearby cells of the same type (e.g., those found within the spatial spread of a single multielectrode array) are highly consistent but it is not known whether this consistency persists across the full extent of cell variability. We therefore measured light responses from a large number of ON-α S RGCs across the full extent of the nasal-temporal axis (Figure 4).

Light stimuli consisted of 1-s flashes that ranged in size from 100 to 1500 μm (see section Materials and Methods). Consistent with much previous work (Pang et al., 2003; Margolis and Detwiler, 2007; van Wyk et al., 2009), response strength varied considerably with stimulus size but was strongest when the size of the stimulus closely matched the size of the dendritic field (Supplementary Figure S4). Thus, for example, the strongest response from a small cell, located at the temporal edge of the retina, arose when the diameter of the flashed spot was 200 μm (Figure 4A, top) while the strongest response from a large cell, found at the nasal edge of the retina, was elicited by a much larger (500 μm) spot (Figure 4A, bottom). Despite the large size difference between the two cells, their responses were quite similar. Indeed, the strongest response of each ON-α S RGC appeared similar across the entire population of cells tested (Figure 4C, *n* = 39). To quantitatively compare responses, we calculated the peak firing rate, the sustained firing rate, the sustained firing rate normalized by the peak firing rate, and, the time to peak and plotted each as a function of the size of the stimulus that produced the strongest response (Figures 4D_1__–__4_). The differences across cells were not statistically significant (*p* = 0.5557, *p* = 0.8660, *p* = 0.5438, and *p* = 0.4263, respectively) suggesting that the peak responses of individual ON-α S RGCs remain similar across the population, despite a wide range of cell sizes.

### AIS Size and Location Influence RGC Excitability

Because scaling was so prevalent in α S RGCs, we sought to understand whether it contributed to cell function. In particular, because the AIS mediates spike initiation, we questioned whether this aspect of scaling influenced the sensitivity of spike generation and propagation. To explore this, we ran a series of computer simulations in which individual neuronal properties of model cells were systematically varied while exploring the effects on (1) spike threshold, (2) the frequency and consistency of spike trains, and (3) the effectiveness with which AIS-initiated spikes triggered somatic spikes.

Seven model cells were created, each from an actual ON-α S RGC for which the entire morphological structure of the cell had been captured (see section Materials and Methods). Dendritic field diameters of the 7 model cells ranged from 231 to 422 μm, approximating the extent of sizes found naturally. In addition to replicating the anatomy of individual cells, the density of voltage-gated ion channels was customized for individual neuronal compartments using previously established values (Fohlmeister et al., 2010) (Supplementary Table S1, see section Materials and Methods). Prior to exploring the role of individual neuronal features, the functionality of model cells was first verified by comparing the kinetics of simulated action potentials to that from physiological measurements (Supplementary Figure S5A, middle column). The close match between simulated and actual spikes suggested the spike generation mechanism in model cells was accurately capturing key elements of normal physiology. In addition, action potentials in model cells were always initiated in the AIS, suggesting that the underlying mechanisms of activation in the model replicated that of normal physiology.

Threshold in model cells was defined as the minimum amplitude of a 1 ms somatic current injection required to elicit an action potential. Individual AIS features were varied systematically and a separate calculation of threshold made for each iteration of each parameter. Because the size of individual features typically varied across the seven model cells, all variations were made relative to the measured feature size in each cell, e.g., the length of the AIS was varied from 50–200% of the nominal (measured) value for that cell. The effect reported is the mean across all seven cells.

**FIGURE 5:**
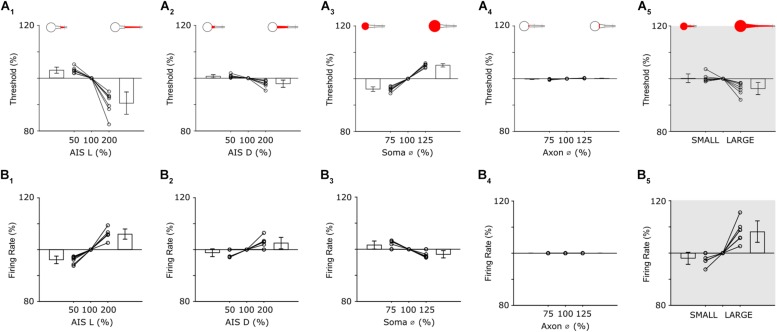
Individual features have distinct effects on neuronal output. **(A_1_)** Threshold as a function of AIS length (all other parameters held constant). Bar plots are the mean threshold changes for all 7 cells at both the minimum (left) and maximum values (right) of AIS length. **(A_2__–__4_)** Similar to **(A_1_)**: AIS distance, soma diameter and initial axon diameter were varied from 50 to 200% (AIS D) or 75–125% (Soma Ø and Axon Ø) of nominal. **(A_5_)** Thresholds with all parameters set to minimum values (SMALL), all parameters set to nominal and all parameters set to maximum (LARGE). **(B_1__–__4_)** Analysis of firing rate when single anatomical parameters (AIS L, AIS D, Soma Ø and Axon Ø) were varied; each line is from a separate cell (*n* = 7). **(B_5_)** Changes in firing rate for the small and large model cells.

Reducing the length of the AIS resulted in higher activation thresholds (Figure 5A_1_); the largest increase occurred when AIS length was set to its minimum value (increase of 2.9 ± 1.1% for a length of 50%). Conversely, increasing the length of the AIS lowered activation threshold with the largest reduction occurred when the length of the AIS was set to its maximum value (8.9 ± 4.2% reduction for an AIS length of 200%). Because the observed variations in AIS length (Figure 2B) are slightly smaller than the 50–200% range tested here, the simulations suggest that the AIS variability arising naturally produces only a modest change in threshold but nevertheless, that AIS length modulates the sensitivity with which the cell responds to a given stimulus. Changes to the distance between the soma and the AIS (AIS D) also influenced threshold but the effect was smaller (Figure 5A_2_, 50%: +0.7 ± 0.6%; 200%: −2.0 ± 1.3%). When threshold was mapped across the full range of AIS lengths and distances (Supplementary Figure S5B), the iso-threshold lines were near vertical, further suggesting the stronger influence of AIS length over distance in modulating activation threshold.

Changes to soma size also influenced sensitivity with reductions in soma diameter leading to lower thresholds (Figure 5A_3_); the smallest diameter tested (75% of nominal) led to a threshold reduction of 4.0 ± 0.9% while the largest diameter (125% of nominal) led to a threshold increase of 5.1 ± 0.6%. Note that the sensitivity to size changes was opposite for soma size vs. that of AIS length (or AIS distance), i.e., larger AISs resulted in lower thresholds while larger somas resulted in higher thresholds. This is not surprising given that larger somas require larger amounts of current injection to depolarize to a given level but nevertheless indicates that uniform increases of size in all cellular features may not result in uniform and predictable changes in threshold levels (see below). The final parameter tested, the diameter of the initial portion of the axon, had only a negligible effect on threshold (Figure 5A_4_, 75%: −0.2 ± 0.1%; 125%: +0.2 ± 0.1%).

To gain insight as to how scaling of all parameters simultaneously might influence thresholds across the population of α S RGCs, we constructed two new models in which all neuronal features (AIS L, AIS D, Soma Ø and Axon Ø) were varied together. The LARGE model had all values set to maximum while the SMALL model used all minimum values. Comparison of the sensitivity of these two cells allowed the effect of comprehensive scaling of all features to be evaluated. Threshold for the SMALL model cell increased by 1.9 ± 1.6% (over nominal) while threshold in the LARGE cell decreased by 3.7 ± 2.3% (Figure 5A_5_). As mentioned above, increases to some neuronal elements reduced threshold while increases to others lowered threshold, e.g., increased soma size raised threshold in the LARGE model while the increased AIS length caused a reduction and therefore, the range of thresholds across the SMALL to LARGE models was smaller than the range induced by some neuronal elements in isolation. This suggests the possibility that scaling may be to help stabilize response sensitivity across the population of a given cell type even across a wide range of feature sizes.

### AIS Properties Influence the Properties of Spike Trains

The second modeling experiments explored the influence of the AIS in shaping the properties of elicited spike trains, i.e., not just the threshold for single spikes. In previous studies, the loss of Na_v_1.6 channels resulted in an inability to produce high-rate spike trains in mouse RGCs (Van Wart and Matthews, 2006). Similarly, in cultured hippocampal pyramidal neurons from newborn mice, the loss of the structural proteins needed to maintain a high density of sodium channels in the AIS reduced the precision of spike timing (Lazarov et al., 2018). To explore the relationship between individual properties of the AIS and properties of the cell’s spike trains, we used the same models and general approach described above but increased the duration of the current injection from 1 to 400 ms, so that multiple spikes would be generated from each stimulus (100 pA).

Analogous to the threshold simulations above, variations in the AIS had small but significant effects on firing rate (Figure 5B). For example, when AIS length was set to 50% of nominal, the mean reduction in firing rate was 3.9 ± 1.4%; doubling the length of the AIS increase firing rate by an average of 6.0 ± 2.0% (Figure 5B_1_). The effects of varying AIS distance were also analogous to the single spike threshold findings with a 50% reduction in AIS D leading to a 1.2 ± 1.5% reduction of firing rate while a doubling of AIS D led to a 2.5 ± 2.2% increase (Figure 5B_2_). Soma diameter also influenced firing rate (Figure 5B_3_) however its effect was smaller than it was for single-spike threshold (75%: +1.6 ± 1.5%; 125%: −1.9 ± 1.4%). The effect was smaller in the single spike experiments because the size of the soma was linearly related to the amount of charge needed to depolarize the cell membrane and thus produce the spike while for the longer duration stimulus in the spike rate experiments, the cell membrane remained depolarized once it was charged and thus less charge was required to generate subsequent spikes. Also similar to the results from the threshold experiments, changes to the diameter of the initial axon had only a minimal effect on firing rate (Figure 5B_4_). After testing the sensitivity of all parameters in isolation, we re-created the SMALL and LARGE models and tested the resultant firing rates (Figure 5B_5_). Interestingly, the increase in firing in the LARGE model (vs. nominal) was 8.2 ± 4.1%, larger than the effect due to any one parameter in isolation and also nearly twice that for single-spike threshold (3.7 ± 2.3%). This larger difference arose because AIS length and AIS distance both had a stronger effect on firing rate while soma size did not and is in contrast to the single-spike threshold results which were strongly affected by soma size (and AIS length). The results, taken together, therefore suggest that the influence of specific neuronal elements as well as the interplay between multiple elements is not constant for all modes of operation. Firing rate in the SMALL model was lowered by 2.0 ± 2.3%, comparable to the 1.9 ± 1.6% reduction for single spike thresholds. It is worth noting that the increase firing rates described in the large model cells were not matched by physiological responses – at least not for light responses (Figure 4). This suggests that other factors, not incorporated into the model, are likely contributing to spike rate (see section Discussion).

### Scaling Influences the Infiltration of AIS Spikes Into the Soma

In the simulations above, variations in the diameter of the initial portion of the axon were found to have little effect on either threshold or pulse rate. What then was the functional role of variability in this part of the neuron? Results from a previous modeling study in neocortical pyramidal neurons suggested that the taper of the proximal axon influences the effectiveness with which AIS-initiated spikes trigger somatic spikes (Mainen et al., 1995). The results of Figure 5A suggest that taper does not directly influence the sensitivity of spike threshold but the shape of the action potential was altered by some of the changes in the previous simulations (Supplementary Figure S5A), including taper, leading us to question the specifics of this effect and, whether the effectiveness of back-propagation was also influenced.

**FIGURE 6:**
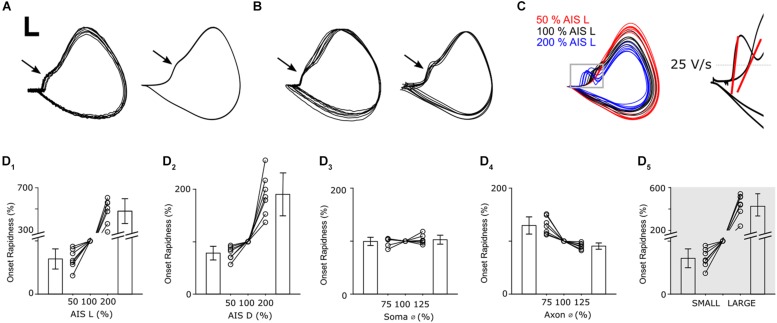
Axonal geometry influences the infiltration of AIS-spikes into the soma. **(A)** Overlay of phase plots of 10 somatic action potentials in a recorded (left) and a modeled (right) RGC. Scale bar: 10 mV/100 V/s. **(B)** Overlay of phase plots (normalized) of one action potential in 10 recorded (left) and 7 modeled (right) cells. (**C**, left) Phase plots of action potentials in one model cell with three different AIS lengths ranging from 50–200% of nominal. (**C**, right) Onset rapidness was defined as the slope of the rate of depolarization when a depolarization of 25 V/s was reached (red lines). **(D_1__–__4_)** Change of onset rapidness when single anatomical parameters were varied. **(D_5_)** Change of onset rapidness when all parameters were either set to their minimum (SMALL) or maximum (LARGE) values.

Plots of membrane voltage (V) vs. the time derivative of membrane potential (ΔV/Δt) are referred to as phase plots and allow the fine details of action potential kinetics to be examined. They are especially useful for comparing specific time segments of the action potential during periods where either V or ΔV/Δt are changing rapidly. Figure 6A shows phase plot overlays of 10 spikes from an actual cell (left) and 10 spikes from a model cell (right). As expected, individual spikes were highly consistent from trial to trial and many aspects of the spike kinetics were matched by the model cell. Overlay of individual spikes from 10 different α S RGCs (Figure 6B, left) revealed high consistency across the population and many of the subtle variations in spike kinetics could be largely replicated in the different model cells (right). Although phase plots report somatic potentials, the AIS-induced spike can be observed in the waveform as the portion of the curve prior to the inflection point (arrows in panels A and B); the inflection point, referred to as the initial segment-somatodendritic (IS-SD) break, occurs at the transition between the initial depolarization arising from the AIS-initiated spike and the onset of the (somatic) action potential (Coombs et al., 1957; Yu et al., 2008) suggesting that the axial current arising from the AIS-initiated spike helps to trigger the somatic spike. The presence of an IS-SD break in all recorded cells is a marker that indicates that spikes are always initiated in the AIS suggesting the process is very reliable in α S RGCs.

As in previous simulations, we varied individual neuronal elements in all 7 model cells and examined the effect on membrane dynamics via phase plots (Figure 6C). The largest differences occurred in response to variations in AIS length and were most noticeable around the IS-SD break with a longer AIS (blue) leading to a sharper rise during the initial phase of the action potential. This leads to a more pronounced IS-SD break for longer AISs while the IS-SD break is almost eliminated for shortened AISs (red). To quantitatively compare changes in phase plots, we calculated onset rapidness (Lazarov et al., 2018), defined as the slope in ΔV/Δt when a rate of 25 V/s was reached; the red lines in Figure 6C (right) depict the onset rapidness calculations for two of the sample traces from the main panel with the one on the left corresponding to an increased AIS length (200%) and the one on the right to a reduced AIS length of (50%). Reduction of AIS length to 50% of nominal led to a decrease of onset rapidness (−33.6 ± 19.0%) whereas doubling led to an increase (479 ± 117%) (Figure 6D_1_). This occurs because the increased length of the AISs results in a stronger inward current during the AIS spike and thus stronger axial currents are delivered to the soma. Changes to AIS distance (Figure 6D_2_) had a similar but less pronounced effect as onset rapidness was reduced by 21.7 ± 13.0% for distance reduction to 50% and increased by 190 ± 41% for a distance of 200%. Soma diameter had only a small influence on action potential shape as onset rapidness changed by less than 3% (Figure 6D_3_, 75%: −0.5 ± 7.8%; 125%: +2.7 ± 8.4%). Thus, in contrast to their relatively small effect on single-spike threshold and firing rate, changes to the initial axon diameter (Figure 6D_4_) had a more substantial effect on spike kinetics with an onset rapidness increase of 30.0 ± 18.3% for a diameter reduction to 75% of nominal and an onset rapidness decrease of 11.7 ± 3.7% when diameter was increased to 125%. Because reducing the size of most elements (relative to that of nominal) resulted in a reduction in onset rapidness while reducing the size of the initial axon diameter resulted in an increase in onset rapidness, these model results suggest that adjustments to the initial axon diameter might be essential to optimize the consistency of the somatic spike both within spikes from the same cell as well as across cells of the same cell type. Use of the LARGE and SMALL models revealed that, similar to the effects with threshold, the simultaneous increase in size of all neuronal elements mitigates the effect of changes to any one parameter change and thus provides further support for the notion that scaling may help to maintain consistency of many response features across a wide range of morphological variations (Figure 6D_5_).

The plots of Figures 6C,D suggest that the shape of the somatic action potential is sensitive to the properties of at least several neuronal elements. The most noticeable change in spike shape was the IS-SD break, suggesting that changes to the neuronal elements in question alter not only the timing of individual spikes but also the strength and effectiveness with which the AIS-initiated spike triggers the somatic spike (Yu et al., 2008). As with the previous modeling experiments, we found here that multiple elements could have an effect and that the polarity could vary for individual elements. Therefore, similar to the experiments with threshold sensitivity, the effect on somatic spike initiation was smaller when multiple parameters were changed simultaneously vs. when the effect of individual parameters. Whereas the diameter of the initial axon (and the corresponding taper of the initial axon segment) had only small effects on threshold and spike rate, it had a more substantial effect on the triggering of the somatic spike, and had the strongest counter-balancing effect to changes in AIS properties. It is of course possible that factors not considered in the model, e.g., the density and/or expression patterns of specific types of voltage-gated potassium channels, could also play a role in normalizing spike thresholds and/or the effectiveness with which the AIS-initiated spike triggers the somatic spike and it will be interesting to explore such effects in future studies.

The change in kinetics of the action potential as a function of AIS length (Figure 6C), suggest that part of the reason for customizing AIS length is to optimize the initiation of the somatic spike. Note that in Figure 6C, the longer AIS (blue) resulted in a faster initial change in voltage (ΔV/Δt) but a lower level in overall depolarization (lower maximum level of V). While our results do not explain why this occurs, it is likely that a ‘too-fast’ delivery of current to the soma is simply not effective for charging the large capacitance of the somatic membrane; it leads to inactivation of some portion of somatic voltage-gated sodium channels with the net result that the somatic action potential has a weaker rise and reaches a lower peak voltage. At the other end of the spectrum, a ‘too-small’ delivery of current (from an AIS that is too short) offers no boost to the somatic spike (no IS-SD break), i.e., the AIS-spike does little to help trigger the somatic spike.

## Discussion

### Summary

Detailed anatomical measurements revealed systematic correlations in size between a wide range of morphological features in α S RGCs of the mouse. For example, cells with large dendritic fields tended to also have large somas. In addition, the AISs of large cells were longer and further from the soma than those of smaller cells. Because the AIS is the portion of the cell that mediates spike initiation, our results suggest that this function is not mediated by a uniform structure across all cells of a given type but instead is sized relative to the rest of the cell. To explore the functional role of this scaling, we developed morphologically- and biophysically realistic computational models that allowed individual aspects of the AIS to be systematically varied. The models suggest that scaling helps to reduce sensitivity variations for the initiation of spikes across the cells of an individual RGC type. This helps to further stabilize the temporal properties of elicited spike trains across the population, and ensure consistency in the conversion of the AIS-initiated spike to a somatic spike. Thus, in addition to a myriad of previously identified pre-synaptic processes that shape the responses of RGCs, here we identify a novel post-synaptic mechanism that completes these other processes. Further, scaling is also likely to contribute to the strong similarities in both the kinetics of individual spikes as well as the spike trains arising in response to light during physiological experiments.

### Somatodendritic and AIS Scaling

Our finding that both soma size and dendritic field diameter increase systematically along the nasal temporal axis in α S RGCs (Figure 1B) differs from an earlier study of these same cells which found that only dendritic field extent (not soma size) increases along this axis (Bleckert et al., 2014). To verify our findings, we performed additional measurements of somatic volume using a detailed 3-dimensional approach in a subset of cells; these measurements confirmed that soma size does indeed vary across cells and that it is strongly correlated to dendritic field size (Figure 1D). The 3-d measurements also revealed that the shape of the soma in α S RGCs was slightly ‘flattened’ along the *z*-axis (perpendicular to the plane of the retina, Figures 1C,E) and thus the 2-d measurements systematically overestimated the actual soma size (Figure 1E). Nevertheless, there was a linear correlation between the two estimation techniques, and thus, taken together, our results show that soma size is indeed variable across the α S RGC population and that it scales with dendritic field size. We found similar levels of scaling in both ON and OFF sub-types of α S RGCs (Figure 1B and Supplementary Figure S2). Scaling in these cells is consistent with previous reports in brisk transient (BT) RGCs of the rabbit retina (Peichl et al., 1987) as well as in midget and parasol RGCs of the primate retina (Watanabe and Rodieck, 1989). While this indicates that scaling is not unique to α S RGCs at least one well studied cell type of the mouse retina does not exhibit scaling (Warwick et al., 2018). At any given location in the mouse retina, ON-α S RGCs were slightly larger than neighboring OFF-α S RGCs (Supplementary Figure S2), analogous to previous reports in ON and OFF midget and parasol RGCs in both human and non-human primate retinas (Dacey and Petersen, 1992; Chichilnisky and Kalmar, 2002).

Previous studies have shown that the properties of the AIS can be variable, both within (Hamada et al., 2016) and across individual cell types (Kuba et al., 2006); such variability is thought to help optimize one or more aspects of neuronal function. These previous findings outside the retina led us to analyze the variability in the AIS properties of α S RGCs and we found that both the length of the AIS as well as its distance from the soma increased as a function of cell size (Figure 3B), i.e., AIS properties were scaled to the overall size of the cell. AIS scaling in α s RGCs is intriguing because it suggests that nature tailors the spike initiating region of each cell based on the size of other cellular features. Interestingly, the relative proportion of the Na_v_ components of the AIS both remained remarkably constant across the entire population (Figure 3). The relative proportion of Na_v_1.6 channels within the AIS (88 ± 4%) was nearly identical in both ON- and OFF-α S cells suggesting that whatever role served by this particular ratio, it is similar in both cell populations. Interestingly, the ratio measured here, from a single cell type in mouse retina, was different from that of a previous study in which the Na_v_1.6 portion comprised only ∼67% of the total length across unspecified RGC types in rat retina (Van Wart et al., 2007). It will be interesting in future studies to learn the functional reason for the ratio difference, and to determine whether the ratio varies for different types of RGCs and/or across different species. Finally, our anatomical measurements revealed that the cross-sectional diameter of the axon at the point from which it emerges from the soma, also increased linearly with cell size. Thus, there was a wider taper that persisted over a longer extent of the proximal-most portion of the axon in larger cells. Because this portion of the axon serves as the direct link between AIS and soma, the fact that it too was scaled provides additional support for the notion that AIS properties are functionally linked to the size of the soma and perhaps the overall size of the cell.

### Scaling and AIS Function

To gain insight into the functional role of the anatomical scaling observed here, we developed a series of morphologically- and biophysically realistic computational models. Each model matched the precise morphology of a single α S RGC that had been dye-filled, stained and captured via confocal imaging. The models allowed individual features of the cell to be systematically varied and thus could provide insight as to the functional role of each. Simulations were run to test all cellular features that been anatomically characterized in the first part of the study (soma size, dendritic field, AIS length, AIS distance and the angular taper of the proximal axon). Not surprisingly, alterations to each of the individual features led to changes in neuronal function, particularly in the initiation and/or propagation of action potentials (Figures 5, 6).

Prior to exploring the role of AIS scaling, we sought to first gain insight into the functional role of the scaling observed between the soma and dendritic field diameter (Figure 1B). In preliminary simulations (not shown), we compared the synaptic input arriving at the soma between two cells that were identical except for the size of their dendritic fields. Because the density of synaptic inputs along the dendrites was the same in both cells, a larger amount of synaptic current could be delivered to the soma of the cell with the larger dendritic field. Further, because soma size was identical in the two cells, the larger amount of synaptic input in the cell with the larger dendritic field produced a stronger depolarization in its soma. Increasing the soma size of the cell with the larger dendritic field (as occurs with scaling) reduces the level of somatic depolarization and thus helps to normalize the peak level of depolarization between the different size cells. This therefore suggests that the scaling between soma size and dendritic field diameter observed here (Figure 1B) may help to normalize responses across the full population of cells and is consistent with the highly similar physiological responses found in neighboring cells of the same type as well as across cells of the same type that are located much further apart (Figure 4).

Simulations that explored the functional role of the AIS revealed that its scaling may also help to normalize responses across the population of α S RGCs. Changes to both the length and the location of the AIS did not alter the absolute level of somatic depolarization but instead modulated the level of somatic depolarization required to elicit an action potential, i.e., spike threshold. Interestingly, the longer and more distal AISs found in larger α S RGCs acted to reduce the threshold for spike initiation, suggesting an opposite relationship for some features, i.e., while increased soma size reduced the level of somatic depolarization (and thus increases threshold), increased length of the AIS as well as the increased distance from the soma (as found in large cells) both acted to reduce threshold. Thus, scaling of multiple features may act to fine tune sensitivity in each cell and ultimately, to normalize sensitivity across the entire population. The ability to fine tune sensitivity via scaling of multiple features might also be particularly attractive during development as it would allow the properties of one feature to be adjusted after other features were already sized, e.g., AIS properties could be fine-tuned after the soma and dendritic field sizes had already been established. The demonstration of plasticity of both the length and location of the AIS (Grubb and Burrone, 2010; Kuba et al., 2010) lends support to this possibility. Our results do not preclude the possibility that other features of the cell, e.g., those not explored in this study, also shape sensitivity as well.

Scaling had an analogous influence on the generation of spike trains with synergistic interactions between different neuronal elements again working to stabilize spike rates. The effects of soma size were again opposed by those of AIS length and distance although soma size had a smaller effect on spike rate (than it did on single spike thresholds). Thus, individual neuronal features do not exert the same influence on all aspects of cell function and the resulting interplay between different elements varies as well. The simulations also revealed that changes to the angular taper of the proximal-most portion of the axon had little effect on either spike threshold or the rate of spiking but instead altered the efficacy with which the AIS-initiated spike triggered the somatic spike. The wider tapers associated with larger cells (Figure 2C), resulted in a slower rise in somatic depolarization (slower onset rapidness, Figure 6D_4_), and thus a smaller IS-SD break. This was in contrast to the effects of longer AISs in large cells, which increased onset rapidness (Figures 6C,D_1_). Thus, for all aspects of neuronal function tested here, increasing the size of some neuronal elements drove the response in one direction while increasing the size of others drove the response in the opposite direction. Taken together, this suggests that slight changes to the size of individual elements can be used to fine tune the performance of a wide range of spiking-related functions. We did not probe the role of onset rapidness or the IS-SD break in detail, but changes to the waveforms (Figure 6C) suggest that if onset rapidness is too steep, the AIS-initiated depolarization of the soma occurs faster than the soma can respond (e.g., faster than the large somatic capacitance can be charged), and thus the somatic spike is delayed; further delays eventually led to failure of the somatic spike. This suggests the rate of increase may influence the efficacy with which the AIS-initiated spike helps to trigger the somatic spike and thus, analogous to the simulations above, the simultaneous scaling of multiple components helps to optimize this aspect of cell function. It is worth nothing that spike kinetics were highly similar across all measured α S RGCs (Figure 6B), even when, for example, axo-somatic and axo-dendritic axons were compared, suggesting that nature tries to keep activation thresholds constant and maintain response consistency across the population of α S RGCs.

### Analysis of Model Sensitivity

Our simulation results were dependent upon the underlying anatomy of the cell as well as the distribution and kinetics of the channels used in the model. As such, we evaluated the sensitivity of the results to many different model parameters to ensure that the results were not being artificially driven (biased) by any one (or few) feature of the model. Anatomy of individual cells closely matched those of actual cells and we chose cells that spanned the full range of sizes found in nature. The consistency of results across this population suggests that the model was not inappropriately sensitive to any one specific cell size or morphological feature. Channel kinetics largely matched those developed by Fohlmeister et al. (2010) and are similar to those used previously by our group. The close match between physiological action potentials and those from the model (Figure 6A and Supplementary Figure S5A) suggests key elements of normal physiology were captured by the model, including the fact that action potentials were always initiated in the AIS in both actual and model cells. Further, similar to physiological measurements across the population of α S RGCs, the action potential kinetics of all model cells were also highly similar, suggesting physiological responses of model cells were not inappropriately sensitive to any specific aspect of the model. Nevertheless, estimates for channel densities in RGCs have varied considerably across previous studies (Fohlmeister et al., 2010; Jeng et al., 2011; Werginz et al., 2014; Guo et al., 2016) and the timing of individual spikes could vary if channel conductances were altered significantly. For example, the incorporation of both low- and high-voltage activated calcium channels into the dendrites of model cells (Margolis et al., 2010) could alter spike timing and thus their effect, especially in relationship to the effect of varying AIS properties, will need to be further investigated, including the possibility that the effects are different for ON vs. OFF sub-types.

### α S RGC Properties Influence the Sensitivity to Artificial Stimulation

Our findings may help to improve strategies for selective targeting of specific RGC types with electric stimulation, e.g., for use in basic research studies or as part of a retinal prosthesis. Previous studies have shown that the AIS is the portion of the RGC that is most sensitive to electric stimulation (Sekirnjak et al., 2008; Fried et al., 2009) and further, that differences in AIS properties can influence sensitivity (Jeng et al., 2011). Thus, our findings that show that at any given retinal eccentricity ON-α S RGCs are slightly larger than OFF-α S RGCs (Supplementary Figure S2), including the fact that the AIS is longer and more distant from the soma in ON cells (Figure 2B), coupled with previous work that found that increases to both AIS length and distance reduce activation threshold in response to electrical stimulation (Jeng et al., 2011), suggests that at any given location ON cells have a slightly lower threshold than do OFF cells. This is important as the ability to selectively target ON vs. OFF pathways would allow natural signaling patterns to be more accurately reproduced with electric stimulation and thus might lead to better quality percepts (Freeman et al., 2011; Werginz et al., 2015; Weiland et al., 2016). Interestingly, previous studies have reported that ON and OFF cells have similar thresholds to electric stimulation (Fried et al., 2009; Tsai et al., 2012), but such studies did not carefully account for comparisons at similar eccentricities.

Note that the ability to capitalize on these small differences in sensitivity will require knowledge of the precise location of each AIS as well as the ability to selectively target each from a nearby electrode; this could be accomplished, for example, with high-density arrays of electrodes along with detailed measurements of the responses from the array (Jepson et al., 2014). Developing strategies that are clinically relevant will also require confirmation that the AIS size differences found here in mouse α S RGCs persist in human RGCs. This may be the case however as it has already been established that ON midget and parasol cells in the human retina have larger somas and dendritic fields, raising the possibility that the AIS scaling found here will persist there as well.

The AIS size difference for ON vs. OFF RGCs could also explain earlier findings in which more complex patterns of electric stimulation were able to differentially activate the two populations (Cai et al., 2013; Twyford et al., 2014). While the mechanism underlying differential sensitivity of the two types has yet to be unequivocally identified (but see Kameneva et al., 2016; Guo et al., 2019), the sensitivity differences between ON and OFF cells persist in the presence of synaptic blockers, suggesting that the differences arise from features intrinsic to the two cell types, most likely within the AIS themselves. The improved understanding of the AIS anatomy found here may lead to a better understanding of the mechanism underlying preferential selectivity and may also help to identify stimulation paradigms that provide even higher levels of selectivity.

## Data Availability Statement

The raw datasets generated for this study are available on request to the corresponding author.

## Ethics Statement

The animal study was reviewed and approved by Institutional Animal Care and Use Committee (IACUC) of the Massachusetts General Hospital.

## Author Contributions

VR, PW, and SF conceived the study, analyzed the results, wrote the manuscript, and prepared the figures. VR performed the immunochemistry and confocal imaging experiments. PW performed the electrophysiology experiments and computational modeling.

## Conflict of Interest

The authors declare that the research was conducted in the absence of any commercial or financial relationships that could be construed as a potential conflict of interest.
