# Supplementary material for: Scaling of the AIS and Somatodendritic Compartments in α S RGCs
Source: Front Cell Neurosci. 2019 Sep 27;13:436. doi: 10.3389/fncel.2019.00436 (PMC6777007; doi:10.3389/fncel.2019.00436)
Supplement: Supplementary file 1 [file Data_Sheet_1.PDF]

Supplementary Material

1 Supplementary Figures

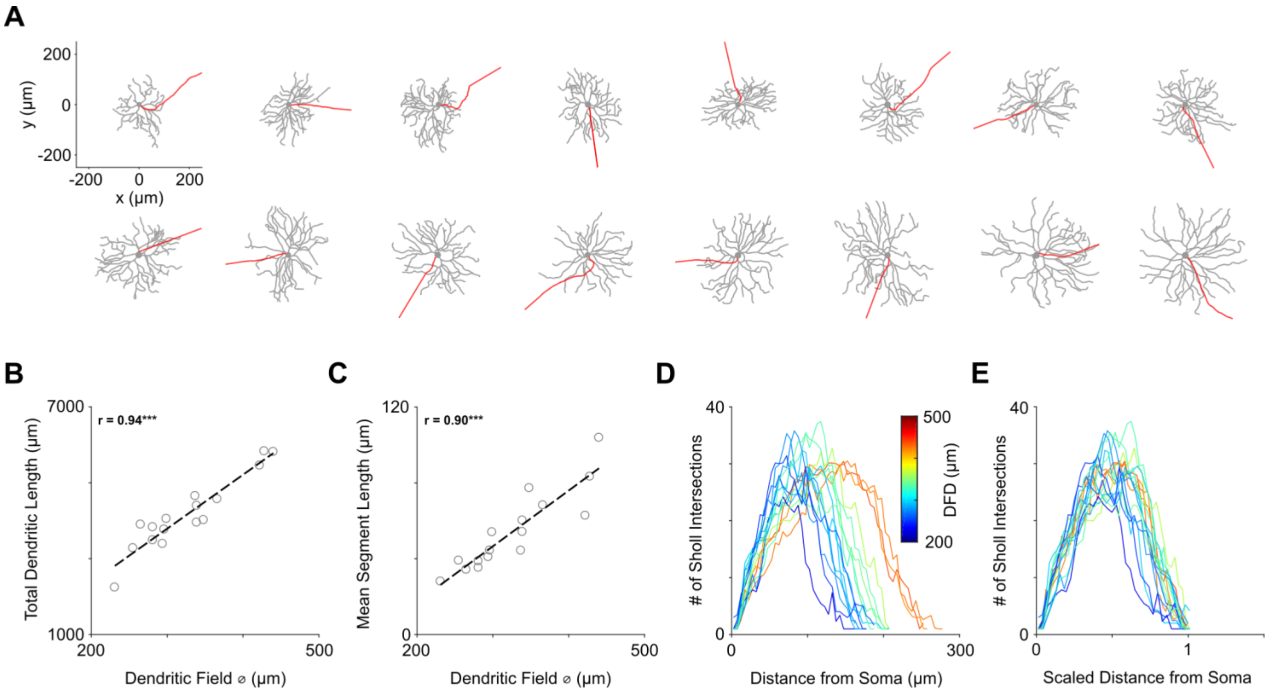

Supplementary Figure 1. Morphological identification of α RGCs. (**A**) Morphologies of 16 traced ON-α S RGCs sorted by dendritic field size (231-433 μm, top left to bottom right). Axons are indicated in red. (**B**) A strong correlation was observed between dendritic field diameter and total dendritic length (p=5x10-8) as well as (**C**) between dendritic field diameter and mean segment length (p=3x10-6). (**D**) The number of Sholl intersections is plotted as a function of the radial distance from the soma. Line color indicates the diameter of the dendritic field. (**E**) Overlay of Sholl plots from (D) with distance from the soma normalized, i.e. radial distances are scaled to the maximum dendritic extent for each cell. Line-colors are the same as in (D).

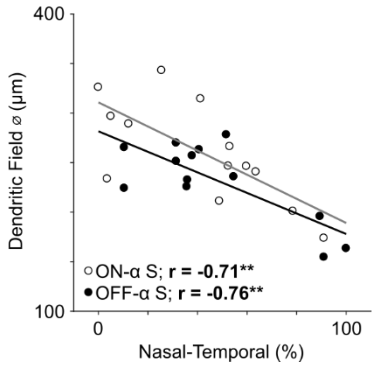

Supplementary Figure 2. ON-α S RGCs are larger at any given location. Each point plots the dendritic field diameter of an α S RGC as a function of position along the nasal-temporal axis. Filled and un-filled points correspond to OFF and ON cells, respectively. Black and gray lines are best-fit linear regressions to filled and unfilled points, respectively.

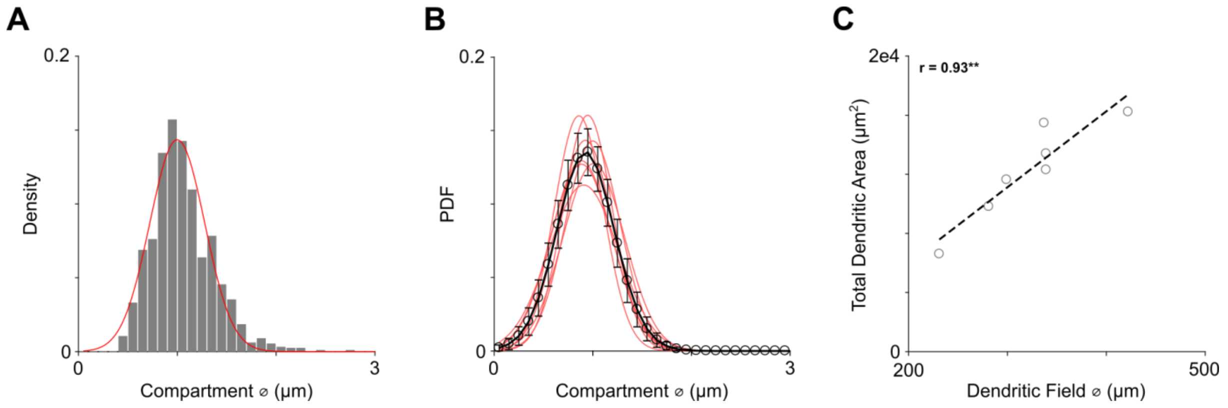

Supplementary Figure 3. Diameters along RGCs dendrites are independent of cell size. (**A**) Tracings of dendritic trees of ON-α S RGCs were divided into segments (compartments) 3-5 µm in length and the dendritic diameters from all compartments of one cell were measured and then plotted as a histogram (grey). A probability distribution function (PDF, red) was fit to the histogram. (**B**) PDFs from all cells (n=7) were overlaid; the black trace is the average across all cells. (**C**) The total dendritic surface area was computed for each cell and plotted vs. dendritic field diameter (p=0.0028).

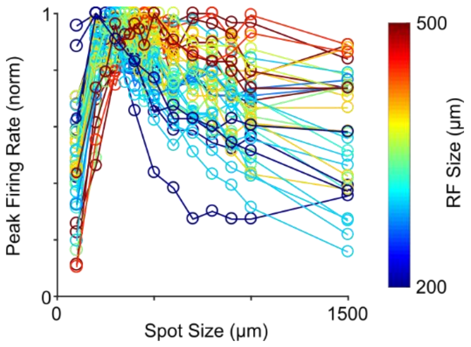

Supplementary Figure 4. Response to light stimulation varies with stimulus size. Each line is the normalized peak firing rate, plotted as a function of stimulus size, for a single cell. Measurements were made for 39 α S RGCs. Line color indicates the spot size that produced the strongest response (Scale at right).

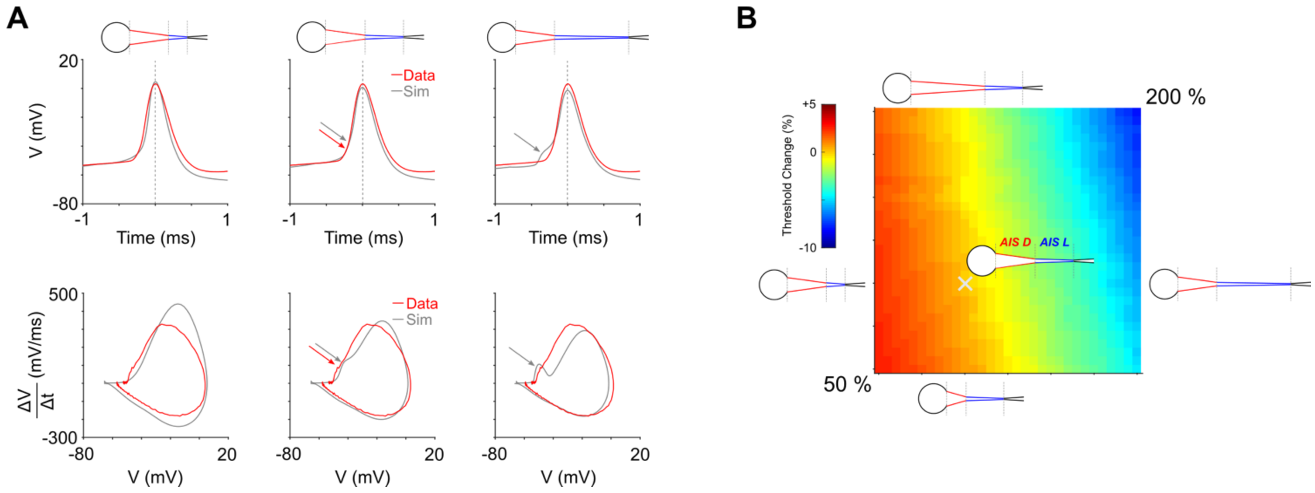

Supplementary Figure 5. Variations in AIS anatomy and the resultant effect on RGC excitability and spike shape. (**A**, top row) Action potential dynamics in model cells for different AIS lengths corresponding to 50 %, 100 % and 200 % of the measured (nominal) AIS length (schematics on top). An experimentally recorded spike is overlaid (red) on all model responses. In simulations with the longest AIS, a pronounced inflection could be observed during the initial rising phase of depolarization (right panel, arrow). (Bottom row) Phase plots, i.e. time derivative of V vs. V, for the action potentials in the top row. Pronounced inflection points in the initial phase of the spikes were observed for longer (nominal and 200 %) AISs (middle and right panel, grey arrows) which could also be observed in spikes measured from the original cell (red arrow). (**B**) Detailed 2-d map of threshold changes (5 % step size) for variation in AIS L (x-axis) and AIS D (y-axis) in one traced RGC. The reference configuration (100 % AIS L and 100 % AIS D) is indicated by the white ‘x’ in the center of the map.

	**Dendrites**	**Soma**	**Soma-AIS**	**AIS**	**Axon**
g_{Na}	60	60	150	420	100
g_{K}	35	35	90	250	50
g_{Ca}	1	0.75	0.75	0.75	0.75
$g_{K,Ca}$	0.17	0.17	0.17	0.11	0.2
g_{L}	0.1	0.1	0.1	0.1	0.1

Supplementary Table 1. Ion channel densities along the membrane of model neurons. Values are based on Fohlmeister et al. (Fohlmeister et al., 2010) with minor modifications. All values are given in mS/cm^2.
